# Diffusion-Controlled Solute and Isotope Transport in the Milk River Aquifer System, Alberta, Canada: Implications for Dating Old Groundwater

**DOI:** 10.1021/acsearthspacechem.5c00397

**Published:** 2026-04-09

**Authors:** Stephanie L. Musy, Roland Purtschert, Neil C. Sturchio, Linnea J. Heraty, Peter Mueller, Jeremy Lantis, Michael N. Bishof, Christof Vockenhuber, Avadhoot Date, Bernhard Mayer, Reika Yokochi

**Affiliations:** † Hydrogeology, Environmental Sciences, 27209 University of Basel, 4056 Basel, Switzerland; ‡ Climate and Environmental Physics, University of Bern, 3012 Bern, Switzerland; § Earth Sciences, 5972 University of Delaware, Newark, Delaware 19716, United States; ∥ 1291 Argonne National Laboratory, Lemont, Illinois 60439, United States; ⊥ Laboratory of Ion Beam Physics, ETH Zurich, 8092 Zurich, Switzerland; # Department of Earth, Energy, and Environment, 2129 University of Calgary, Calgary, Alberta T2N 1N4, Canada; ∇ Geophysical Sciences, 2462 University of Chicago, Chicago, Illinois 60637, United States

**Keywords:** isotope hydrology, matrix diffusion, aquitard−aquifer exchange, groundwater dating, krypton-81, chlorine-36

## Abstract

Krypton-81 (^81^Kr) and chlorine-36 (^36^Cl) are among the few isotopic tracers capable of constraining groundwater residence times on 10^5^–10^6^ year timescales. In sedimentary aquifer systems bounded by low-permeability units, however, diffusive solute exchange can strongly modify tracer distributions and bias apparent ages derived from concentration ratios. In the transboundary Milk River Aquifer (MRA), progressive chloride enrichment caused by diffusion across shale aquitards complicates the interpretation of ^36^Cl/Cl as a chronometer. Here, we combine new measurements of ^81^Kr, ^36^Cl, stable chlorine isotopes (^37^Cl/^35^Cl), and ^14^C with advection–diffusion transport modeling to quantify the importance of matrix diffusion on tracer systematics and inferred groundwater ages. The simulations reproduce the observed decrease in ^36^Cl/Cl and concomitant increase in δ^37^Cl along regional flow paths, demonstrating that diffusive influx of Cl-rich aquitard water dominates the evolution of the chlorine isotope system. In contrast, modeled and observed ^81^Kr activities show substantially lower sensitivity to diffusive exchange over the timescales considered. A comparison of simulated and measured tracer relationships indicates that, in the MRA, apparent ages derived from ^36^Cl primarily reflect chloride addition rather than radioactive decay, whereas ^81^Kr provides a more robust and conservative chronometer for fossil groundwater. These results highlight the value of integrating stable and radioactive chlorine isotopes with noble gas dating and explicit transport modeling to disentangle decay from transport effects. The approach developed here provides a quantitative framework for interpreting multitracer data sets in regional aquifers affected by long-term diffusive exchange and has broader implications for assessing fossil groundwater resources in similar hydrogeological settings.

## Graphical abstract

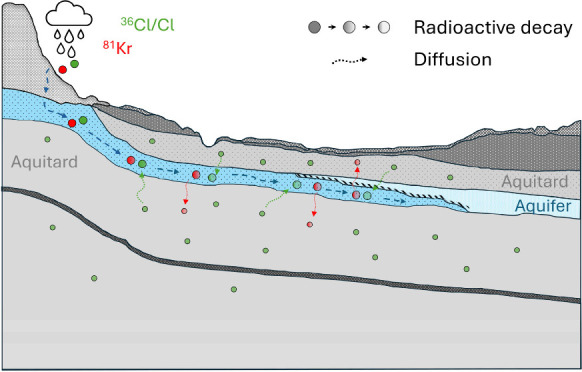

## Introduction

Groundwater represents the planet’s largest accessible freshwater reserve and sustains nearly half of global drinking and agricultural demands.^1–3^ In many sedimentary basins, however, a substantial portion of this resource is “fossil” groundwater, recharged thousands to millions of years ago, that is effectively non-renewable on human time scales.^1,4,5^ Understanding the residence time, origin, and flow behavior of such water is essential both scientifically and socially: it provides insight into Earth’s long-term hydrological and climatic history, while guiding sustainable management of aquifers increasingly relied upon for domestic and agricultural water supplies especially during droughts.^3–6^

The Milk River Aquifer (MRA), spanning southern Alberta (Canada) and northern Montana (U.S.A.; Figure 1), exemplifies the challenge of managing groundwater resources in semi-arid transboundary regions. Extending over 26 000 km^2^, this Late Cretaceous sandstone aquifer provides a crucial water supply for rural communities, agriculture, and industry across a semi-arid transboundary region.^7,8^ However, historical records indicate that intensive pumping since the midtwentieth century has caused persistent declines in hydraulic heads, confirming ongoing “groundwater mining”,^9^ that is, abstraction rates exceeding natural recharge.^10,11^ Understanding groundwater residence times and flow dynamics in this system has therefore become central to developing long-term water management policies.^12^

**Figure 1:**
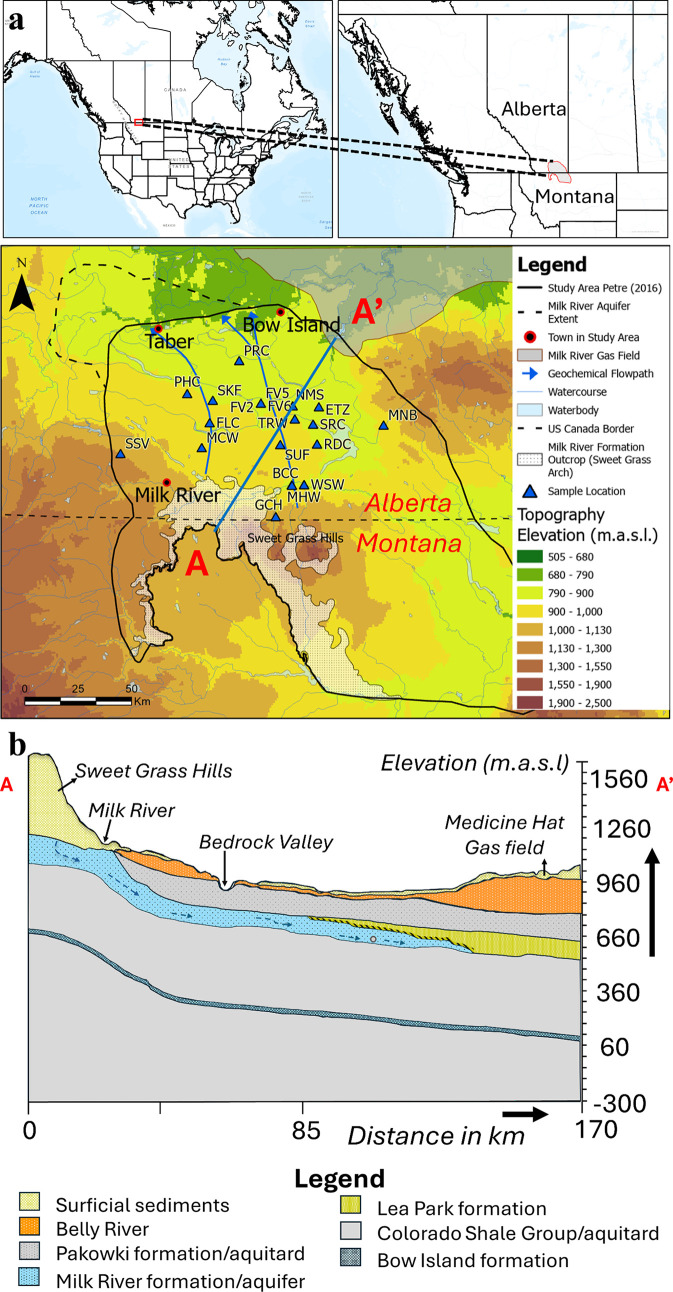
(a) Upper maps show the location of the Milk River aquifer in northern Montana, U.S.A., and southern Alberta, Canada. The lower map shows topography, aquifer recharge locations, sample locations, and geochemically based flowpaths. The line A–A′ indicates the location of the cross-section. (b) Schematic cross-section showing the generalized stratigraphic sequence that includes the Milk River Formation.

Determining the residence time of very old groundwater requires chronometers with half-lives on the order of several hundred thousand years. The cosmogenic radionuclides chlorine-36 (^36^Cl, *t*_1/2_ = 301 kyrs) and krypton-81 (^81^Kr, *t*_1/2_ = 229 kyrs) are uniquely suited for this purpose. These radioactive isotopes extend groundwater dating well beyond the range of radiocarbon (^14^C, *t*_1/2_ = 5.7 kyrs) and serve as independent tracers for waters aged between 10^5^ and 10^6^ years, enabling reconstructions of recharge history, paleoclimate influences, and regional flow patterns.^13–17^ In the following, the term groundwater age refers to an integrated measure of mean residence time, defined as the average time that water parcels spend in the subsurface between infiltration and sampling or discharge at springs.^18–21^

Chlorine-36 has been used for several decades as a tracer of residence times in fossil groundwaters.^13,22,23^ Advances in accelerator mass spectrometry (AMS), particularly the use of large tandem accelerators, allow effective suppression of molecular and isobaric interferences (notably ^36^S), enabling measurement of very low ^36^Cl/Cl ratios (down to ∼10^–15^) and corresponding ^36^Cl concentrations.^24^ Because AMS analyses require only a few milligrams of chloride, groundwater sample volumes are typically small (often <100 mL), depending on chloride concentration. Unfortunately, ^36^Cl, or more precisely the ^36^Cl/Cl ratio used in practice, is sensitive to spatially and temporally varying atmospheric input parameters, and to the subsurface addition of chloride from aquitards or connate waters, potentially obscuring the radioactive decay signal.^13,22,23,25–27^ In contrast, ^81^Kr, as a chemically inert, noble gas with negligible subsurface production under most conditions and a nearly uniform initial abundance in space and time in the atmosphere,^28^ is assumed to be a robust tracer of fossil groundwater.^15,28–33^ The early application of ^81^Kr was constrained by the analytical demands of measuring it using low-level counting techniques^29^ or accelerator mass spectrometry.^34,35^ Substantial progress was achieved in the development of magneto-optical atom trap technology (ATTA), first demonstrated by a group at Argonne National Laboratory (U.S.A.).^36–38^ This innovation reduced the required water sample volumes by several orders of magnitude, while improving the sensitivity and precision of measurements, enabling the feasibility of ^81^Kr dating for groundwater and glacial ice.^16,39–43^ Beyond its role as an independent and conservative chronometer, ^81^Kr can be useful for constraining parameters needed to interpret other age tracers, including cosmogenic ^36^Cl and radiogenic ^4^He.^16,17,39,44–47^ Even chemically conservative tracers such as ^81^Kr can be influenced by physical processes that complicate age interpretation. In particular, groundwater mixing and diffusive exchange within and across aquifers and their confining units can modify tracer distributions and bias apparent residence times.^26,43,48–53^ The ^36^Cl system is especially sensitive to these effects because chloride is highly mobile and can diffuse from adjacent formations, which commonly leads to overestimated apparent ages if such inputs are not accounted for.^13^ ^81^Kr may also be affected, in rare and specific geological settings, by *in situ* production.^32^

From a modeling perspective, the challenge lies in representing the coupled effects of advection, dispersion, diffusion, radioactive decay, subsurface production, and isotopic fractionation. While lumped parameter models (LPM^54–56^ ) offer a simplified baseline, reactive transport frameworks that explicitly include all these processes provide a more realistic interpretation of isotopic tracer data.^48,57–61^ The MRA, with its thick, confining shales and localized fracture networks, exemplifies a system in which this complexity must be resolved for accurate groundwater age interpretation.^7,11,12,59^

The earliest detailed study of the MRA was conducted in the late 1950s and focused on its geology and hydrology.^10^ Subsequent studies documented systematic spatial variations in the chemical and isotopic characteristics of MRA groundwater, with downgradient increases in salinity, δ^2^H, and δ^18^O values.^13,15,26,62–68^ During the 1980s, the International Atomic Energy Agency (IAEA) coordinated a research program to evaluate the radioactive isotope tracer methods available at the time for determining the residence times of MRA groundwaters. Most results of this IAEA program were published in a special issue of *Applied Geochemistry* (summarized by Fröhlich et al.^14^). Tracer studies showed that ^14^C was detectable only within approximately 20–30 km of the recharge area, limiting its applicability to a small portion of the aquifer. In contrast, ^36^Cl/Cl ratios decreased systematically downgradient, reflecting both radioactive decay and, more importantly, dilution by chloride derived from aquitards or connate sources.^64^ However, the relative contributions of these processes and their spatial extent have remained poorly constrained, leading to large, and in some cases contested, uncertainties in inferred groundwater residence times. Hydrodynamic modeling suggested lower residence time limits of approximately 0.25 Myrs, while ^36^Cl/Cl ratios (uncorrected for ^36^Cl dilution by addition of ^36^Cl-free chloride from shales) gave upper limits of 2 Myrs. A single ^81^Kr measurement yielded an apparent age of approximately 140 kyrs, consistent with independent hydraulic and uranium isotope–based estimates (0.1–0.6 Myrs), thereby underscoring the potential of ^81^Kr as a robust tracer for very old groundwater. However, analytical constraints at the time precluded the analysis of additional ^81^Kr samples.^14^

To account for the observed variability in tracer concentrations and activities within the MRA, several mechanisms have been proposed, including macroscopic dispersion, restricted recharge areas, membrane filtration effects, and matrix diffusion across aquitards.^13,26,67,69–72^ Critical evaluations of these hypotheses by Hendry et al.^64^ and by Fröhlich et al.^14^ concluded that aquitard matrix diffusion provided the most consistent explanation for the available geochemical and isotopic data. Subsequent conceptual and numerical modeling studies incorporated additional processes such as cross-formational flow and fracture permeability.^7,12^ However, these models did not explicitly quantify the impact of diffusive exchange on tracer systematics, leaving its influence on groundwater age interpretations incompletely constrained.

Despite decades of isotopic and hydrogeological investigation, several key uncertainties persist regarding the interpretation of tracer concentrations and groundwater ages in the MRA. The foremost challenge lies in quantitatively representing diffusive mass exchange across aquifer–aquitard interfaces. Existing numerical models only partially capture the influence of matrix diffusion and advective–diffusive transport on the spatial evolution of chloride concentrations and ^36^Cl/Cl ratios. Consequently, the relative contributions of solute influx from the adjacent aquitard versus internal low-permeability units within the MRA remain poorly constrained. Stable chlorine isotope ratios (^37^Cl/^35^Cl) provide a valuable, independent constraint on diffusive solute transport because diffusion induces measurable isotopic fractionation of chloride, even in the absence of distinct source signatures. Despite its diagnostic value, stable chlorine isotope data for the MRA have not been reported previously. Their inclusion allows direct evaluation of diffusion-controlled chloride exchange between the aquifer and its confining units, thereby offering a means to test and refine transport models that have so far relied solely on concentration-based evidence. Further methodological uncertainty concerns the integration of ^36^Cl and ^81^Kr within a unified transport and age-dating framework. The initial ^36^Cl/Cl input ratio remains difficult to calibrate owing to both variable cosmogenic input near the recharge area and the potential addition of chloride from subsurface sources. These parameters are commonly treated as fixed or weakly constrained, although their uncertainty propagates directly into inferred residence times. Finally, discrepancies between isotopic and hydraulic residence times highlight unresolved aspects of regional flow and recharge dynamics. Collectively, these gaps underscore the need for a fully coupled isotopic–hydrodynamic modeling approach that explicitly links diffusion, isotope systematics, and flow processes, and that explicitly accounts for uncertainty in key input parameters. Such integration is critical not only for reconciling age discrepancies and refining solute transport models, but also for ultimately designing appropriate use policies for this transboundary fossil groundwater resource.

To address these uncertainties, we combine a new suite of isotopic measurements (including ^81^Kr, ^36^Cl, ^37^Cl/^35^Cl, and ^14^C) with a two-dimensional advection–diffusion model to quantify the role of matrix diffusion in shaping tracer distributions and apparent groundwater ages in the MRA. The isotope data set constrains present-day tracer concentrations along eastern and western flow paths, while ^37^Cl/^35^Cl values provide an independent indicator of diffusive chloride exchange across aquifer–aquitard boundaries. Numerical simulations implemented in HydroGeoSphere^73,74^ explicitly represent advection, dispersion, diffusion, and radioactive decay, and explore parameter uncertainty through Monte Carlo analyses. Comparison of observed and simulated tracer–tracer and tracer–distance relationships allows us to quantify diffusion-induced dilution of ^36^Cl, evaluate the potential bias in ^81^Kr-derived residence times, and delineate the conditions under which each tracer yields robust age information. This combined observational–modeling framework enables us to (i) quantify diffusion-controlled solute exchange, (ii) reassess long-standing interpretations of ^36^Cl systematics in the MRA, and (iii) evaluate the robustness of ^81^Kr as a chronometer for very old groundwater in regional aquifers affected by long-term diffusive exchange.

### Study Site

Most previous geochemical studies of the MRA focused on a roughly 100 × 100 km square area extending north from the U.S.A.–Canada border. The generalized stratigraphy of the Cretaceous bedrock in the study area is shown in a cross-section that extends from approximately 20 km south of the Sweet Grass Hills, Montana (U.S.A.), to approximately 40 km east of the town of Bow Island, Alberta (Figure 1). The MRA is contained in a sequence of marine sandstones interbedded with gray shales within the 90 to 145 m thick Milk River Formation.^7,26,72^ This formation is confined between two thick shale aquitards, except where it outcrops around the Sweetgrass Hills area near the U.S.A.–Canada border (Figure 1). The abundance of sandstone within the Milk River Formation decreases gradually toward the northern end of the study area. The Milk River Formation conformably overlies a sequence of bentonitic, dark gray to black marine shales of the Colorado Group, which has a total thickness of 500 to 650 m.^64^ In the lower Colorado Group, there are thin sandstone layers, including the Bow Island sandstone. The Milk River Formation is unconformably overlain by the Pakowki Formation, which consists of gray bentonitic shales with thin bentonite layers. The Pakowki Formation is approximately 120 m thick in the northeastern part of the study area and becomes thinner to the west.^7,8^ Above the Pakowki Formation are mixed aquifer–aquitard sedimentary units of the Foremost Formation.^75,76^ Unconformably overlying the Foremost Formation is a thin layer (approximately 2 m) of Quaternary glacial deposits.^7^

The hydraulic conductivities of the Pakowki Formation and the Colorado Group shales, which confine the Milk River Formation, range from 10^–10^ to 10^–14^ m s^–1^.^26,77^ The MRA dips gently northward, eastward, and westward from a recharge zone located around the Sweet Grass Hills, resulting in a radial flow pattern.^7,10,67,78^ Regional flow is directed downgradient toward the north, with the Milk River acting as a major hydraulic discharge boundary that intercepts much of the northerly groundwater flux.^7^ Transmissivity values within the aquifer vary spatially, ranging from 10^–6^ to 10^–4^ m^2^ s^–1^ in southern Alberta^10^ and up to 10^–2^ m^2^ s^–1^ near the Sweet Grass Hills in Montana, where localized fracturing enhances secondary permeability.^79,80^ Recharge occurs primarily through outcrops near the Sweet Grass Hills. Estimated effective recharge rates are on the order of 5–10 mm year^–1^, representing less than 3% of annual precipitation.^7^ In confined portions of the aquifer, artesian conditions persist locally, though hydraulic heads have declined substantially since the midtwentieth century due to intensive groundwater abstraction.^10,11^

## Experimental Methods

Groundwater samples were collected during two sampling campaigns in October 2021 and May 2022. Details of sampling and analytical methods for each measured parameter are given below. No unexpected or unusually high safety hazards were encountered.

### Chloride (Cl^–^) Concentrations

Chloride concentrations were measured in unfiltered water samples by ion chromatography (Dionex ICS-2100) at a flow rate of 1 mL min^–1^, using a Dionex IonPac AS20 4 mm separation column and AG20 4 mm guard column. KOH eluent injection began at 5 mM for 15 min, followed by a gradient of 3.3 mM min^–1^ to a final concentration of 55 mM that was held for 1 min. The instrument was calibrated using a series of dilutions of a NIST-traceable 7-ion standard solution. The relative standard deviation of reported concentration values is ±2.2%.

### Chlorine Stable Isotope Ratio

Chlorine (Cl) has two stable isotopes, ^35^Cl and ^37^Cl, with respective proportions of 75.8 and 24.2%.^81^ The chlorine isotopic ratio ^37^Cl/^35^Cl is used to study the origin of solutes and track pollutants,^82–86^ quantify diffusion in aquifers,^87,88^ and trace the history of magmas.^89–91^ For the Cl stable isotope ratio measurements, dissolved chloride was precipitated as AgCl from aliquots of unfiltered water samples upon addition of excess AgNO_3_ followed by overnight ripening of precipitates in a dark cabinet. The resulting AgCl was recovered by centrifugation, washed with dilute HNO_3_, dried, and reacted in a sealed borosilicate glass tube with excess CH_3_I at 300 °C for 2 h to produce CH_3_Cl. The produced CH_3_Cl was purified by molecular-sieve gas chromatography, cryofocused from He carrier gas, and then admitted to a Delta-V Plus isotope-ratio mass spectrometer (Thermo Fisher Scientific, Waltham, MA, U.S.A.) and analyzed in dual-inlet mode by measurements at *m*/*z* 50 and 52.^92,93^ Measured ^37^Cl/^35^Cl ratios are reported in conventional delta (δ) notation relative to the international reference material standard mean ocean chloride (SMOC).^94,95^

δCl37⁡(‰)=[(Cl37Cl35)sample(Cl37Cl35)SMOC−1]×1000 1

The δ^37^Cl values of chloride were normalized against analyses of the NaCl isotopic reference material ISL-354 (δ^37^Cl_SMOC_ = +0.05‰).^92,96^ Reference samples were prepared by dissolving ISL-354 in ultrapure water, then treated the same as the groundwater samples. Analytical uncertainty of δ^37^Cl values is ±0.1‰.

### Chlorine-36

^36^Cl (*t*_1/2_ = 301 kyr) is a cosmogenic radionuclide that has been widely applied to date old groundwater, ice, and sediments over timescales of approximately 30 to 1500 kyr.^17,23,97–104^ In groundwater studies, ^36^Cl is typically evaluated using the ^36^Cl/Cl ratio rather than absolute concentrations.^23,105^ The chloride mass balance as a function of groundwater residence time *t* (years) can be expressed as^22,23^

2 $$R_{m}C_{m}=R_{0}C_{0}e^{-\lambda_{36}t}+R_{\mathrm{eq}}C_{0}\left(1-e^{-\lambda_{36}t}\right)+\left(C_{m}-C_{0}\right)R_{\mathrm{ex}}$$

where *R* denotes ^36^Cl/Cl ratios and *C* denotes chloride concentrations. Subscripts m and 0 refer to measured and initial values at recharge, respectively. If all parameters in eq 2 are known, a model ^36^Cl age (*t*) can be derived from the measured isotope ratio and chloride concentration. The evolution of the ^36^Cl system is governed by the three terms in eq 2: (i) the initial ^36^Cl ratio at infiltration (*R*_0_ = ^36^Cl_0_/Cl_0_), which decreases over time due to radioactive decay; (ii) the ingrowth toward the secular-equilibrium production ratio within the aquifer (*R*_eq_); and (iii) the isotopic composition of externally supplied chloride from the subsurface (*R*_ex_). In the simplest case, when external chloride inputs and *in situ* production are negligible, the system reduces to pure radioactive decay, allowing a straightforward age estimate. Table 1 summarizes the parameters considered in the analytical model shown in Figure 2.

**Table 1:** ^36^Cl/Cl Parameters Assumed for the MRA in eq 2 and Figure 2 ^a^

parameter	western flow path	eastern flow path
^36^Cl/Cl at recharge (*R*_0_)	8.00 × 10^–13^	8.00 × 10^–13^
secular ^36^Cl/Cl in the aquifer (*R*_eq_)	4.00 × 10^–15^	4.00 × 10^–15^
^36^Cl/Cl of added Cl in the subsurface (*R*_ex_)	1.00 × 10^–14^	1.00 × 10^–14^
initial Cl concentration at recharge (mg L^–1^) (*C*_0_)	40	15
subsurface accumulation rate (mg L^–1^ year^–1^)	0.0004	0.0002

a *R*_ex_ corresponds to values reported by Fröhlich et al.^14^.

**Figure 2:**
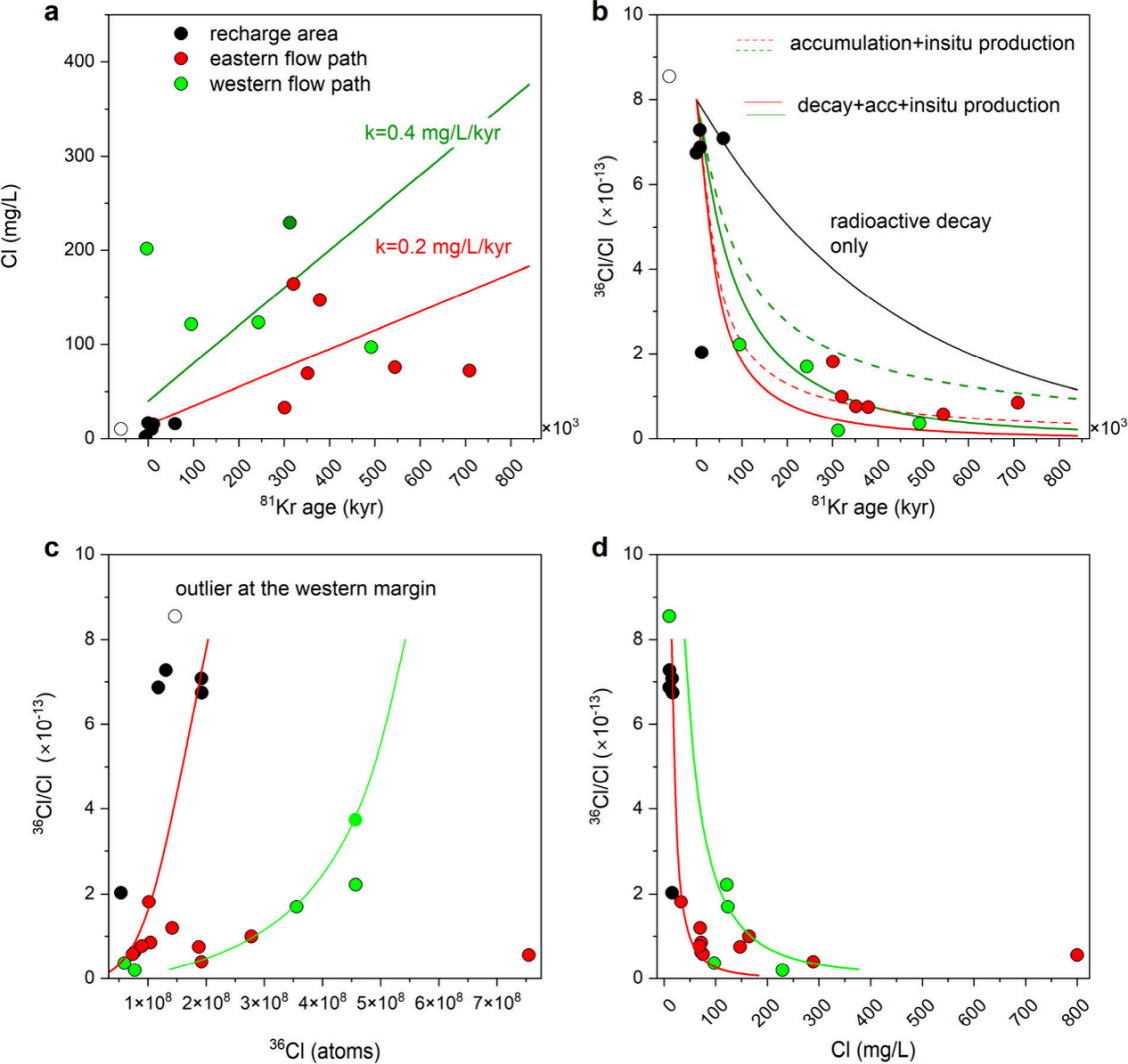
(a) Cl concentration and (b) ^36^Cl/Cl as a function of the ^81^Kr decay ages, (c) ^36^Cl/Cl as a function of the number of ^36^Cl atoms, and (d) ^36^Cl/Cl as a function Cl concentration. The points represent the resampled wells in the recharge area (black) and in the eastern (red) and western (green) parts of the study area. The open symbol represent the well SSV at the western margin of the study area (Figure 1). The model lines account for critical parameters for ^36^Cl/Cl dating, as discussed by Phillips et al.,^13^ including the ^36^Cl/Cl ratios at recharge, at secular equilibrium in the aquifer, and the added Cl in the subsurface. Over the relevant timescales considered here, Cl concentrations at recharge may also vary.

In this study, water samples were taken in 1 L PVC bottles. ^36^Cl/Cl ratios were measured at the Laboratory of Ion Beam Physics (ETH, Zurich) using accelerator mass spectrometry (AMS). Cl was extracted from the water samples, sulfur was removed, and chloride was precipitated as silver chloride. The measurements were performed on the 6 MV EN Tandem Accelerator, extracting negative Cl ions from the silver chloride samples, applying mass analysis and molecular destruction, and finally separating the isobar ^36^S in the gas-filled magnet and gas ionization detector.^105^ The measured ^36^Cl/^35^Cl ratios were blank-corrected and normalized to the internal standard K382/4N.^106^

### Radiokrypton (^81^Kr and ^85^Kr)

Krypton has six stable isotopes, of which ^84^Kr is the most abundant, accounting for approximately 57% of natural krypton.^30,107^ Of the 11 known radioactive krypton isotopes, only ^81^Kr and ^85^Kr have half-lives suitable for investigating environmental processes on hydrologically relevant timescales.^108^ ^81^Kr (*t*_1/2_ = 229 kyr) is produced naturally in the atmosphere by cosmogenic reactions, whereas ^85^Kr (*t*_1/2_ = 10.76 years) atmospheric activity is derived primarily from nuclear fuel reprocessing and serves as a tracer of modern groundwater components.^109,110^ In contrast to other gas tracers, such as ^3^H/^3^He, SF_6_, or ^4^He, radiokrypton is largely insensitive to recharge conditions (temperature, pressure, excess air) and to common subsurface processes, including degassing or fractionation. This robustness arises because radiokrypton dating relies on isotope ratios of the same element (i.e., ^81^Kr/Kr and ^85^Kr/Kr), which are relatively unaffected by physical gas partitioning processes.^16,46,108,111,112^ After recharge, radioactive decay is therefore the dominant process controlling radiokrypton evolution. Under the simplifying assumption of piston-flow transport, groundwater residence time (*t*) can be calculated as

3 $$t=-\frac{1}{\lambda }\ln\left(\frac{R_{\mathrm{sample}}}{R_{0}}\right)$$

where *R*_sample_ is the ratio measured in the sample and *R*_0_ is the atmospheric ratio at infiltration.

Despite the extremely low natural abundances of ^81^Kr and ^85^Kr (subparts per trillion relative to total krypton in air), recent advances in atom trap trace analysis (ATTA) have enabled precise measurements of these isotopes.^38,41,109^ ATTA methods now allow dating of groundwater over age ranges of approximately 30–1300 kyr using ^81^Kr,^38,44^ and 5–50 years using ^85^Kr^20,21,111^ with water sample volumes on the order of 20–40 L, corresponding to approximately 1–2 μL of extracted krypton.^113^

In this study, 100–200 L of water were degassed in the field using a semipermeable membrane contactor apparatus for subsequent ^81^Kr and ^85^Kr measurements (device B from Yokochi^108,114^ ). Separation and purification of Kr from dissolved gas samples were carried out at the University of Chicago and Bern using several gas chromatographic steps.^115^ Measurements of ^81^Kr and ^85^Kr were made using a magneto-optical atom trap with the ATTA-3 system at Argonne National Laboratory.^38,116^ Results for ^81^Kr are reported as percent modern krypton (pMKr), defined relative to the modern atmospheric ratio (^81^Kr/Kr = 9.3 × 10^–13^),^116^ while ^85^Kr activities are reported in decays per minute per cubic centimeter of krypton at standard temperature and pressure (dpm cc_Kr_^–1^).

### Radiocarbon (^14^C)

Carbon-14 (^14^C, *t*_1/2_ = 5730 years) has been one of the most widely used radiometric tracers since its introduction in the late 1940s, supporting chronologies in archeology, geology, hydrology, and other disciplines for materials up to roughly 40–45 kyr old.^117–119^ In the atmosphere, ^14^C is continuously produced through cosmogenic reactions and is incorporated into the dissolved inorganic carbon (DIC) of infiltrating meteoric waters, thereby entering groundwater systems.^117^ Once in the subsurface, ^14^C provides information on recharge timing and paleoclimate for waters recharged on timescales of approximately 0.2–30 kyr.^118,120^ Its application is particularly valuable in arid and semi-arid regions, where modern recharge is limited, and groundwater withdrawals increasingly draw upon older, non-renewable, or shallow fossil water resources.^121–126^

^14^C sampling was done with 100 mL glass bottles sealed with a butyl septum stopper, which had been prepared with AgNO_3_ to prevent biological activity. In the field, the evacuated bottles were submerged in a flow of sample water and filled through a syringe needle, thus preventing any atmospheric contact with the sample water. The ^14^C samples were measured at the Laboratory of Ion Beam Physics (ETH, Zurich) using the Mini Carbon Dating System (MICADAS).^127,128^ Conventionally, groundwater ^14^C activity is expressed as the fraction of a modern, prenuclear reference activity (*A*_REF_ = 13.5 dpm g_C_^–1^), reported as percent modern carbon (pMC).^129,130^

Applications of ^14^C to groundwater dating are typically limited by geochemical reactions that modify dissolved inorganic carbon (DIC) at recharge and along the flow path.^48,117,131^ The main complication arises from subsurface carbon sources that introduce ^14^C-free DIC, thereby diluting the atmospheric signal. Numerous correction models have been developed to account for such geochemical dilution effects and to reconstruct the initial ^14^C activity expected from atmosphere–water exchange.^119,129,131–136^ However, at the timescales relevant to this study, these dilution processes are negligible, and no correction was applied to the measured ^14^C activities.

### Numerical Model

This study employed a two-dimensional transient numerical model to simulate groundwater flow and reactive solute transport under a range of synthetic hydrogeological scenarios. Simulations were carried out using HydroGeoSphere (HGS^73^), an integrated surface–subsurface hydrological model (ISSHM) capable of solving variably saturated flow and transport processes in a fully coupled framework.^57,74,137,138,138–142^ Only the porous and fracture domains, and the solute transport modules were used in this study, following the simplified configuration used in Musy et al.^48^ Transport simulations included explicit modeling of advective-diffusive transport and radioactive decay for ^81^Kr, ^14^C, as well as three chlorine isotopes (^36^Cl, ^35^Cl, and ^37^Cl). Total chlorine (Cl) is the sum of ^35^Cl and ^37^Cl.

#### Parameter Sensitivity and Uncertainty

Monte Carlo simulations were conducted to explore the sensitivity of model outcomes to uncertainty in key parameters. A normal distribution was defined around the parameter(s) of interest, and values were randomly sampled for each iteration. The model was then run using the sampled parameter set. Sampled parameters are presented below in terms of their mean and distribution bounds (i.e., mean ± range). The standard deviation was set to 30% of the full distribution range. Parameters that are not subject to uncertainty analysis were held constant across all simulations.

#### Domain Definition

The model represents an idealized 2D vertical cross-section designed to capture the key hydrogeological features of an aquifer–aquitard system. The domain was defined as a single porous medium with spatially variable hydraulic properties (Figure S1a in Supporting Information). It spans 120 km in length (*x* direction), 1500 m in height (*z* direction), and 1 m in width (*y* direction). A laterally continuous aquifer, centered at a depth of 750 m, was embedded within a low-permeability matrix representing the aquitard (Figure S1 in Supporting Information). Aquifer thickness was set to 90 m. The domain was discretized using a finite difference scheme with horizontal and lateral spacings of 100 m (d*x*) and 1 m (d*y*), respectively. Vertical discretization (d*z*) was non-uniform: cell thickness ranged from 16 cm near the aquifer to 40 m near the domain boundaries, with exponential refinement toward the aquifer to accurately resolve concentration gradients and diffusive fluxes.

#### Flow and Transport Boundary and Initial Conditions

No-flow and zero-flux transport boundary conditions were applied to all model limits, except at the upgradient (*x* = 0 km) and downgradient (*x* = 120 km) faces, where fixed hydraulic heads were imposed to maintain a constant horizontal hydraulic gradient of 0.4% (Figure S1). This configuration established a steady lateral flow field through the confined aquifer. Hydraulic conductivity was set to 30 m year^–1^ in the aquifer and 3 × 10^–4^ m year^–1^ in the surrounding aquitard, representing a contrast of several orders of magnitude. Porosity was assigned based on literature values: 10% in the aquifer and 7% in the aquitard. These values align with those reported in the literature.^59,77^ Under these conditions, the average Darcy groundwater velocity along the aquifer flow path (i.e., *V*_*x*_) was approximately 13 cm year^–1^. The longitudinal dispersivity was set to α_L_ = 350 ± 200 m, with values varying across scenarios. In all cases, transverse dispersivity (α_T_) was set as a constant fraction of the longitudinal value, with α_T_ = 0.1α_L_. The diffusive transport is governed by the effective diffusion coefficient *D*_eff_ (m^2^ year^–1^), defined as the product of the free-phase diffusion coefficient *D*_free_ (m^2^ year^–1^) and the tortuosity factor τ. While *D*_free_ values were held constant for each solute or tracer (see Table 2), uncertainty in *D*_eff_ was introduced by sampling τ from a normal distribution centered at 0.1 and ranging between 0.01 and 1. To assess the importance of diffusive effects, an additional simulation was set up using the average parameter set except for all hydrodynamic dispersion coefficients, longitudinal and transverse dispersion (α_L_ and α_T_) as well as molecular diffusion (*D*_free_), artificially set to zero.^143^

**Table 2:** Tracer Infiltration Concentrations, Initial Concentrations, and Free-Phase Diffusion Coefficients (*D*_free_)^49,52,134,144–147^ in the HGS Numerical Model

tracer	infiltration concentration	initial concentration^a^	free phase diffusion coefficient (m^2^ year^–1^)
^81^Kr	105 ± 10 pMKr	0 pMKr	0.045727
^14^C	100 pMC	0 pMC	0.0378
Cl	15 ± 10 mg/L	2000 ± 1000 mg/L	0.0315
^36^Cl/Cl × 10^–15^	700 ± 200	8 ± 4	*D*_36_ = *D*_35_ = 0.0315
δ^37^Cl	–2.5 ± 0.5‰	0.5 ± 0.5‰	*D*_37_ = 0.0314 = *D*_35_/1.0016

a For^81^Kr and ^14^C, the initial concentration refers only to the aquitard, as aquifer concentrations were inherited from the final state of the preceding model run. For the chlorine isotopes (^36^Cl, ^35^Cl, and ^37^Cl), the initial concentrations were applied uniformly across the entire model domain.

#### Numerical Implementation

The model was run in transient mode to ensure a quasi-steady state for both flow and transport processes. To achieve this, the simulations proceeded in two stages: (1) the flow model was run independently for 50 000 years, and (2) the coupled flow and transport model was then run for an additional 1 000 000 years. This approach allowed tracer concentrations to reach secular equilibrium under the imposed boundary conditions. For ^81^Kr and ^14^C, transport simulations were always initialized using the concentration fields from the final state of the previous simulation. The concentration at the infiltration time was 105 ± 10 pMKr and 100 pMC for ^81^Kr and ^14^C, respectively. The adopted initial ^81^Kr value reflects the slightly overmodern activities measured in the youngest recharge-proximal samples (e.g., GCH, MCW, and SSV; Table 3), which also exhibit detectable ^14^C activities. The small deviation from the modern atmospheric value likely represents analytical uncertainty^28,42,108^ and/or minor subsurface effects,^32^ which are considered negligible for the purposes of the present modeling. In contrast, for ^36^Cl, ^35^Cl, and ^37^Cl, both initial concentrations and infiltration source concentrations were sampled from distributions defined in Table 2, reflecting their greater variability. While underground production of ^36^Cl is generally considered a minor contributor in this setting, it was not explicitly modeled due to computational constraints (see the Results and Discussion section). To account for this uncertainty, initial ^36^Cl concentrations in the aquitard were set to positive values and varied across simulations.

**Table 3:** Tracer Observations in the Milk River Aquifer

ID site	distance from recharge (km)	flowpath	^36^Cl/Cl × 10^–15^	err. ^36^Cl/Cl × 10^–15^	Cl (mg L^–1^)	δ^37^Cl (‰)	^85^Kr (dpm cc_Kr_^–1^)	^81^Kr corrected (pMKr)	^14^C (pMC)	^81^Kr PF corrected age (kyrs)	err. ^81^Kr PF corrected age (kyrs)	^14^C PF age (kyrs)	err. ^14^C PF age (kyrs)
uncertainty	±2					±0.1	±1	±2	±0.03				
BCC	26.6	S	687	32	9.1		<0.2	107	1.5	8	6	35	0.4
BCH (house)	26.5	S	728	37	10.2		<0.3	108	1.2	7	6	36	0.4
EHF	27.3	E			12.7		<0.2	91	0.6	64	5	42	0.8
WSW	27.5	S	675	20	15.3		1.95	92	0.6	60	6	42	0.8
MHW	27.4	S	708	23	14.7	–1.99			0.7		0	41	0.7
GCH	13.7	S	203	17	16.1	–2.33	<0.2	106	13.0	12	6	17	0.1
KRW	11.9	S			2.8		<0.2	112	11.7	–6	6	18	0.1
MCW	60.7	W			208	0.36	<0.2	111	0.4	–3	6	45	4.6
FV5	66.4	E	63	6	68.5	–0.91			0.6		0	43	0.8
FV6	66.4	E	58	6	74.2	–1.14		21	0.2	544	12	52	2.6
FV2	66.4	E	86	15	69.5	–1.52		13	0.2	709	651	52	12.3
ETZ	64.6	E	77	6	66.9	–1.14		38	0.7	352	24	41	2.6
MNB	69.9	E	39	6	285	–0.91			0.2		0	52	12.4
NMS	63.7	E	56	9	921	0.62			0.3		0	48	1.6
SKF	75.7	W	170	8	120	–0.89	2.6	53	0.1	243	6	54	17.8
TRW	57.5	E	75	7	95.7	0.85		35	0.2	379	9	53	15.0
SRC	56.1	E	119	10	49.9	–1.02			0.5		0	44	1.0
RDC	47.4	E	181	11	30.1	–2.65	<0.2	44	0.2	301	11	52	12.3
PKW	55.3	E			48.5						0		
SUF	45.8	E	100	7	52.8	–0.24		42	0.4	320	9	46	1.2
SSV	89.8	W	854	24	8.8		1.9	132	21.8	–60	5	13	0.1
SST	89.7	W			19.4						0	0	
FLC	67.5	W	222	11	116		<0.3	82	0.2	95	6	53	15.5
PHC	84.6	W	20	5	237			43	0.1	312	8	58	28.1
PRC	88.2	W	36	5	100			25	0.1	492	21	57	25.5

Time integration was handled using HGS variable time-stepping scheme, employing the Crank–Nicolson implicit finite-difference method. The Newton–Raphson solver was used to solve the nonlinear system of equations.

## Results and Discussion

### Tracer Systematics

The newly acquired groundwater tracer data are summarized in Table 3, together with the residence-time estimates derived from simple piston-flow decay models using ^81^Kr and ^14^C. With the exception of the new ^81^Kr measurements, which were not previously available, the overall tracer patterns are consistent with those reported in earlier studies^14,15,64^ and are therefore summarized only briefly. Sampling locations were spatially referenced using QGIS,^148^ and distances from the recharge area in the Sweet Grass Hills were calculated along the inferred primary flow direction (Table 3). The recharge reference point was defined based on topographic and geological considerations (Figure 1) with an estimated positional uncertainty of ± 2 km. To account for potential differences in hydrodynamic evolution and boundary conditions, samples were grouped according to their position east or west of the recharge zone, reflecting the bifurcation of regional groundwater flow paths.

The distance from the recharge area is calculated from the reference point identified based on topographic and geological evidence (coordinates shown in Figure 1). ^81^Kr activities are corrected for modern air contamination based on ^85^Kr measured activities (see main text for details). Groundwater ages based on ^81^Kr piston-flow (PF) modeling are calculated assuming a recharge activity of 110 pMKr. Radiocarbon (^14^C) PF ages are uncorrected for geochemical processes affecting ^14^C values. Errors for ^81^Kr, ^85^Kr, and ^14^C represent average analytical uncertainties.

#### Radiocarbon and Krypton Isotope Data (^14^C, ^85^Kr, and ^81^Kr)

Measured ^81^Kr activities decrease systematically along the regional flow path, from slightly above the atmospheric reference value of 100 pMKr near the recharge area to 22, 32, and 44 pMKr in the most distal wells (FV6, FV2, and PHC, respectively). Activities of ^85^Kr are generally below the analytical detection limit (0.2–1 dpm cc_Kr_^–1^), indicating the absence of a modern groundwater component and confirming that no significant air contamination occurred during sampling (e.g., through leaky well casings). Three samples (FV2, ETZ, and, to a lesser extent, SKF) contain detectable ^85^Kr, which is attributed to minor contamination by modern atmospheric krypton with an activity concentration of approximately 70 dpm cc_Kr_^–1^.^110^ For these samples, the modern krypton contribution was subtracted prior to calculating ^81^Kr-derived residence times.

Using the corrected ^81^Kr activities, apparent piston-flow decay ages (eq 3) range from a few thousand years near recharge to approximately 709 kyr in the distal parts of the aquifer (Table 3). These ages are consistent with uncorrected ^14^C activities calculated assuming an initial value of 100 pMC, all of which lie at or beyond the effective dating limit of radiocarbon (i.e., 30 kyr), confirming the limited applicability of ^14^C for most of the MRA.

#### ^36^Cl/Cl Ratio (^36^Cl/Cl)

Figure 2 illustrates the temporal evolution of chloride concentrations (Figure 2a) and ^36^Cl/Cl ratios (Figure 2b) as a function of ^81^Kr decay age. Chloride concentrations are lowest near the recharge area and increase irregularly with residence time. Samples from the western flow path exhibit a higher apparent chloride accumulation rate than those from the eastern flow path, with mean values of approximately 0.4 and 0.2 mg L^–1^ kyr^–1^, respectively (Figure 2a). If the accumulation rate *k* is assumed to be spatially and temporally constant, the excess chloride relative to recharge (*C*_m_ – *C*_0_; eq 2) can be approximated by *kt*, where *t* is the ^81^Kr decay age.

Under this assumption, the evolution of the ^36^Cl/Cl ratio can be examined as a function of residence time, chloride concentration, and total ^36^Cl inventory (Figure 2c and d). In Figure 2b, dashed curves represent expected ^36^Cl/Cl ratios assuming chloride accumulation alone and secular-equilibrium production in the aquifer and aquitards (Table 1), whereas solid curves additionally account for radioactive decay. The comparison shows that the observed decline in ^36^Cl/Cl is dominated by continuous chloride addition rather than by radioactive decay, rendering ^36^Cl unsuitable as an independent chronometer in the MRA. This observation underscores the importance of identifying and quantifying the mechanisms responsible for chloride input to the aquifer.

A mechanism proposed in earlier studies is the diffusion of chloride from Cl-rich aquitard units into the aquifer.^14,26,64,65^ Using the diffusion parameters listed in Table 2, a tortuosity τ = 0.1, an aquifer thickness *L* = 90 m and observed accumulation rates of 0.2–0.4 mg L^–1^ kyr^–1^, the thickness Δ*y* of the aquitard layer contributing to a diffusion-controlled chloride flux can be estimated using Fick’s law (eq 4). Assuming a chloride concentration contrast Δ*C* ≈ 2000 mg L^–1^ between aquitard and aquifer (Table 2), this back-of-the-envelope calculation yields Δ*y* values of approximately 23–230 m. These values are consistent with the thickness of the confining units (Figure 1) and support diffusive exchange with adjacent aquitards as a plausible mechanism for the observed chloride accumulation.

$$\frac{dC}{dt}=k≅\frac{\Delta C}{\Delta y}\frac{D_{\mathrm{eff}}}{L}\Rightarrow \Delta y=\frac{\Delta C}{k}\frac{D_{\mathrm{eff}}}{L}$$ 4

#### Stable Chlorine Isotopes (δ^37^Cl)

Measured δ^37^Cl values range from −2.7 to +0.9‰ and increase systematically with rising chloride concentrations (Table 3 and Figure 3). This trend is inconsistent with ion filtration models^71^) but is consistent with Fickian diffusion along a concentration gradient. A logarithmic function provides a better description of the δ^37^Cl–Cl relationship than a binary mixture curve (Figure 3), supporting diffusion-driven fractionation rather than simple mixing between isotopically distinct chloride sources as the dominant control on ^37^Cl values.

**Figure 3:**
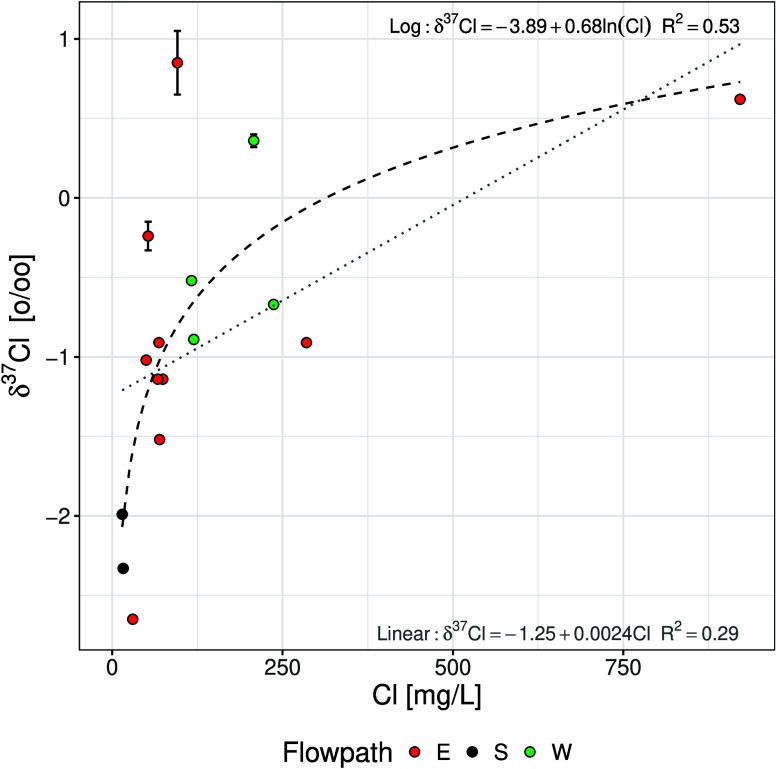
Observed δ^37^Cl values (‰) vs Cl concentration (mg L^–1^) for groundwater samples from the Milk River aquifer. The dotted line represents binary mixing of the two endmembers (aquifer and aquitard pore waters) identified in Table 2, and the dashed line is a logarithmic (*R* ^2^ = 0.53) best fit to the data set. Observations are shown as black (recharge area), green (western flowpath), and red (eastern flowpath) markers, based on their location.

At low chloride concentrations, δ^37^Cl values reflect an ^37^Cl-depleted meteoric recharge endmember. Measurements of inland precipitation in western and central Canada indicate δ^37^Cl values between −3.5 and −1.2‰, with an amount-weighted mean of −2.3‰,^149^ consistent with the lowest δ^37^Cl values observed near recharge. At higher chloride concentrations, groundwater progressively acquires chloride diffusing from the Colorado Group shales. Bulk shale-derived chloride is characterized by δ^37^Cl values of approximately +0.5‰, assuming an initial seawater value (0.0‰) modified by preferential diffusive loss of ^35^Cl over geological history (Table 2). However, the δ^37^Cl signature transferred to the aquifer during diffusion is not solely controlled by this source composition. Because ^35^Cl diffuses slightly faster than ^37^Cl, kinetic isotope fractionation accompanies diffusive transport, resulting in preferential enrichment of ^37^Cl in the residual source in the shales and a slightly lower δ^37^Cl signature in the diffusive flux entering the aquifer, but still high enough to result in increasing δ^37^Cl values in the aquifer. Such behavior has been demonstrated in both field studies and laboratory experiments.^144,150^ In the MRA, upward and lateral diffusive fluxes from these shales therefore provide a high-chloride, ^37^Cl-enriched end-member consistent with the isotopic composition observed in distal wells.

#### Link to ^81^Kr Interpretation

The strong influence of aquitard-derived chloride exchange on the ^36^Cl system raises the broader question of whether analogous diffusive mass-transfer processes could also affect other tracers. Although ^81^Kr is chemically inert and its subsurface production is negligible, it may still undergo physical exchange across aquifer–aquitard boundaries. If such an exchange were significant on timescales of 10^5^–10^6^ yrs, it would bias ^81^Kr-derived residence times toward older apparent ages. A central objective of this study is therefore to quantify, using numerical transport modeling, the extent to which diffusive coupling between the aquifer and surrounding shales influences ^81^Kr systematics. The δ^37^Cl observations provide independent support for diffusion-driven solute exchange, strengthening the basis for evaluating whether similar physical transport processes affect noble-gas tracers.

### Simulation Outputs

After 1 000 000 years of coupled flow and transport simulation using average parameter values, the modeled tracer distributions exhibit distinct, diagnostic spatial patterns across the aquifer cross-section (Figure 4). ^81^Kr shows a moderate decrease from an input activity of approximately 105 pMKr at recharge to approximately 65 pMKr at *x* = 120 km (Figure 4a), reflecting its long half-life. In contrast, ^14^C activities decline rapidly along the flow path and become negligible beyond 30–40 km from recharge (Figure 4b), reflecting its much shorter half-life and indicating that radioactive decay dominates its evolution over the simulated timescales.

**Figure 4:**
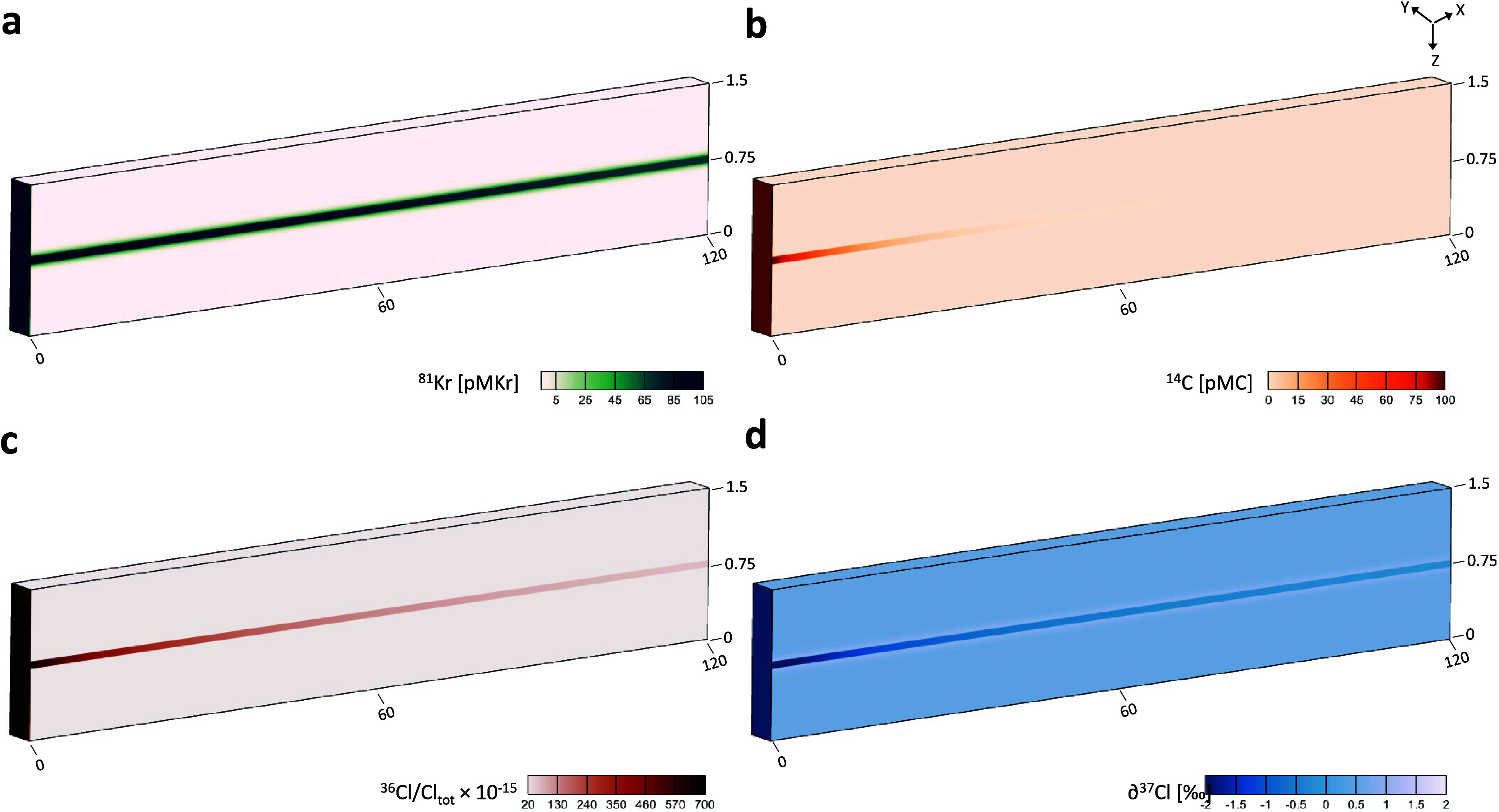
Simulated tracer distributions after 1 000 000 years of coupled flow and transport modeling with the average values of the parameters. (a) ^81^Kr concentrations in pMKr, (b) ^14^C concentrations in pMC, (c) ^36^Cl/Cl ratios, and (d) δ^37^Cl values in ‰. The model dimensions are in km.

The simulated ^36^Cl/Cl ratios decrease markedly downgradient, reaching values below 62 × 10^–15^ at *x* = 120 km (Figure 4c). This decrease is substantially faster than that of ^81^Kr despite their comparable half-lives, consistent with the combined effects of radioactive decay and diffusive addition of chloride with low ^36^Cl/Cl from adjacent aquitards. Simulated δ^37^Cl values increase from approximately −2.5‰ at recharge to about −0.5‰ at the downgradient end of the aquifer (Figure 4d). This value remains slightly lower than the bulk aquitard δ^37^Cl value of approximately +0.5‰, consistent with diffusion-controlled exchange under finite timescales. The development of an isotopic “halo” along the aquifer–aquitard interfaces is also characteristic of diffusion-driven isotope fractionation.^144,150,151^

### Comparison of Simulated and Observed Tracer Profiles

To evaluate model performance, simulated tracer concentrations were extracted along a horizontal profile at the aquifer mid-depth (*z* = 750 m). Ensemble means and ±2σ uncertainty envelopes from 50 Monte Carlo realizations are shown in Figure 5, together with results from a diffusion-free reference scenario. For radioactive tracers, this reference case corresponds to purely advective-dispersive transport with radioactive decay; for δ^37^Cl, it yields no isotopic fractionation.

**Figure 5:**
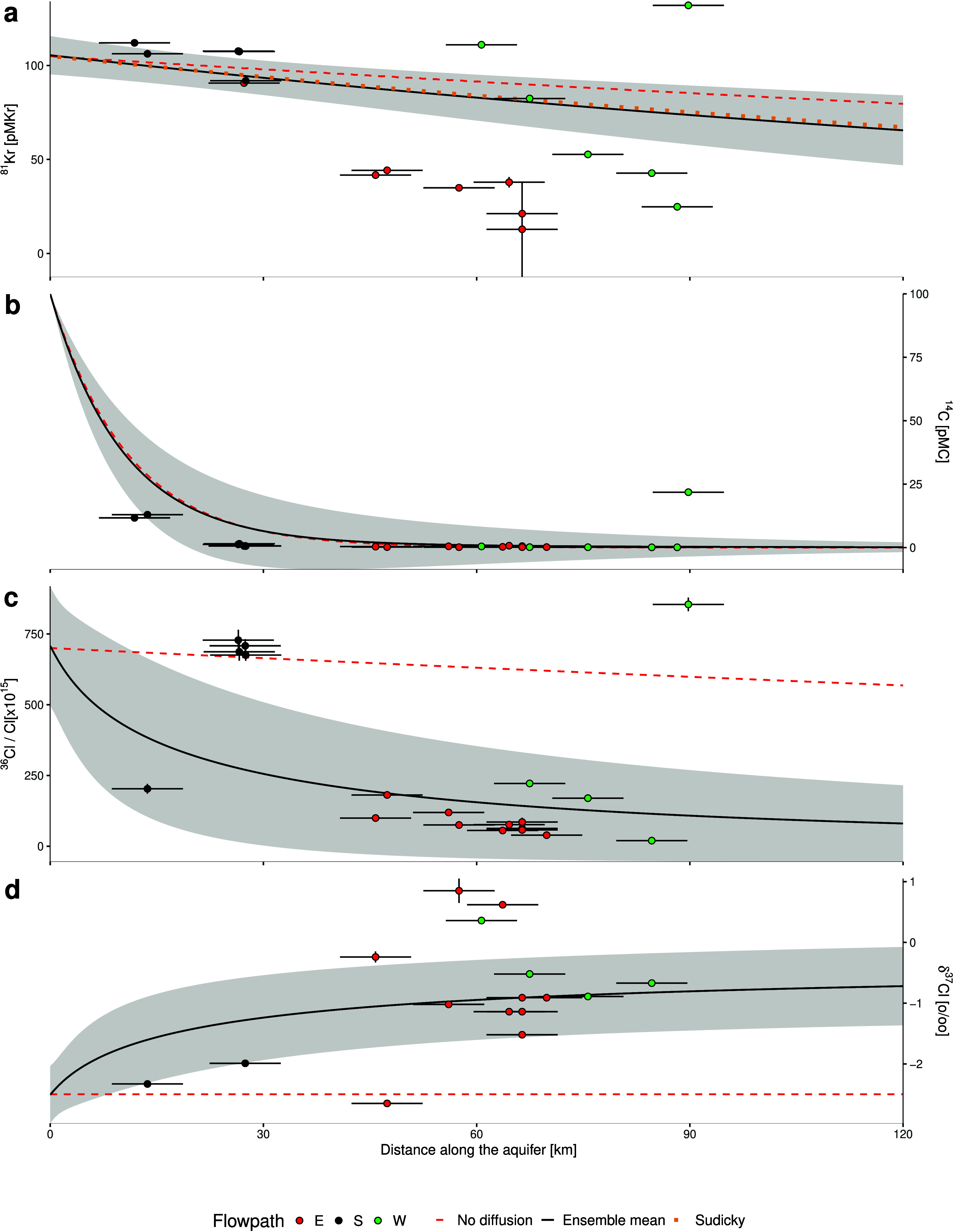
Comparison between simulated and observed tracer data along the Milk River Aquifer. Panels show (a) ^81^Kr, (b) ^14^C, (c) ^36^Cl/Cl × 10^–15^, and (d) δ^37^Cl. Modeled concentrations are extracted along a 2D profile centered within the aquifer (*z* = 750 m). The black line is the model ensemble mean (corresponding to the simulations using the average parameter set, shown in Figure 4). The shaded area represents ±2 standard deviations across all 50 realizations with random parameters within the distribution. Observations are shown as black (recharge area), green (western flowpath), and red (eastern flowpath) markers, based on their location. Distances were calculated in QGIS relative to a recharge point in the Sweet Grass Hills (see Figure 1 for location). The red dashed line represents the simulation using the average parameter set, except for the dispersive and diffusive parameters, which are set to 0. The dotted orange line shows results calculated using the analytical Sudicky diffusion model^52^ with the same average parameter values.

Radiocarbon is the most sensitive tracer to decay because of its short half-life and becomes effectively undetectable beyond 30–40 km from recharge. Comparison with the diffusion-free reference scenario indicates that diffusive transport has a negligible influence on ^14^C over the timescales considered, thereby justifying the use of uncorrected ^14^C activities and the omission of geochemical dilution effects associated with interaction with ^14^C-depleted subsurface carbon reservoirs.

In the absence of diffusion, ^81^Kr and ^36^Cl/Cl would be expected to evolve similarly along the flow path. Near recharge, the diffusion-free scenario reproduces observations reasonably well, likely reflecting the thinner confining units and enhanced vertical connectivity due to shallow fracturing. Farther downgradient, however, diffusive exchange becomes increasingly important. At *x* = 120 km, diffusion accounts for approximately 90% of the observed reduction in ^36^Cl/Cl, compared with approximately 19% for ^81^Kr. Most measured ^36^Cl/Cl values fall within the model uncertainty envelope, indicating that the simulations adequately capture the dominant diffusive chloride input controlling ^36^Cl systematics (see also Figure 2). The agreement between simulated and observed δ^37^Cl increase provides independent evidence that diffusion is the governing transport mechanism.

Observed ^81^Kr ages display greater spatial scatter, with western samples generally appearing younger than eastern ones at comparable distances. This pattern may reflect heterogeneity in the aquifer’s hydraulic conductivity, whereby groundwater in the western flow path travels more rapidly than in the eastern path. For ^36^Cl/Cl, such hydraulic variability would be partially masked by the larger diffusive chloride flux, yielding a low ^36^Cl/Cl ratio along the western flow path, which in turn produces a correspondingly larger age bias discussed earlier. Subsurface production of ^81^Kr could, in principle, contribute to variability, but this would require unusually high concentrations of uranium or other parent nuclides, which is unlikely in the MRA. Reported uranium concentrations are on the order of 1.4 ppm in the aquifer and 3.2 ppm in the confining aquitards, with thorium concentrations of 3.9 and 11.7 ppm, respectively,^59,152^ values typical of sedimentary rocks and far below those associated with significant subsurface radionuclide production.^32^ Additional contributing factors may include 3D flow pathways not represented in the 2D model domain, spatially distributed or multiple recharge areas, and mixing effects associated with well construction (e.g., differing screened interval lengths), which could broaden residence-time distributions in individual samples.

### Comparison of ^36^Cl and ^81^Kr Apparent Ages

Figure 6 compares simulated and observed tracer behavior in both activity–activity and apparent piston-flow age–age space. Near recharge, observations cluster around the diffusion-free piston-flow reference line, indicating minimal diffusive influence. With increasing distance, both tracers diverge progressively from this reference, reflecting the cumulative effect of chloride addition from aquitards. This divergence is reproduced by the ensemble of simulated trajectories, with most observations falling between the mean model trend and the piston-flow reference. This comparison demonstrates that apparent ages derived from ^36^Cl increasingly reflect transport-driven chloride addition rather than radioactive decay, whereas ^81^Kr retains a closer link to groundwater residence time over the same distances.

**Figure 6:**
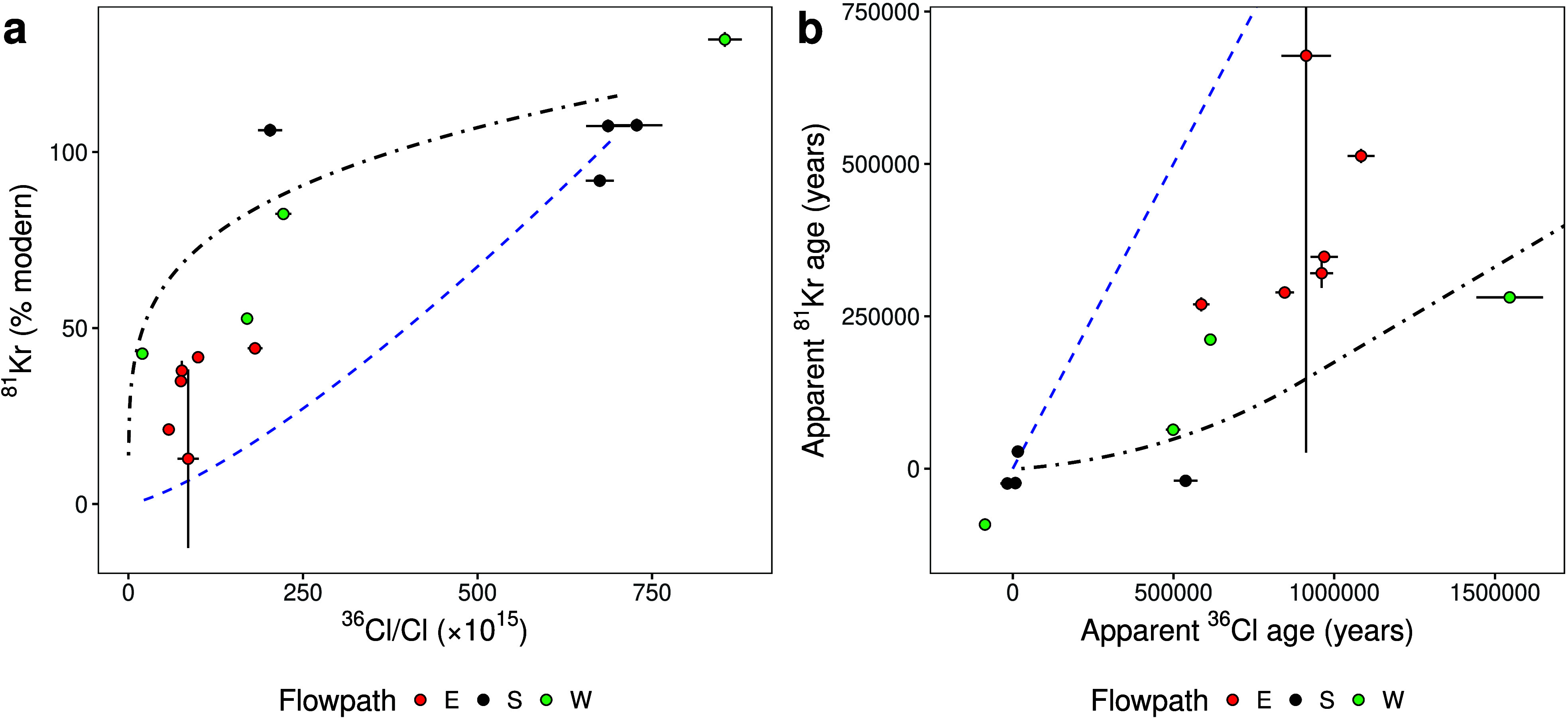
Comparison of modeled and observed ^36^Cl/Cl and ^81^Kr behavior in (a) activity–activity space and (b) apparent piston-flow age–age space. Observations include propagated analytical uncertainties. The blue dashed line represents the piston-flow reference corresponding to the diffusion-free simulation. The black dot-dashed line shows the extended model trend derived from the HGS ensemble simulations. In panel a, the ensemble mean relationship was extrapolated to lower activities using a log–log linear regression fitted to the simulated tracer activities. In panel b, a generalized additive model fitted in log–log space was used to represent the nonlinear relationship between apparent ages, and the resulting trend was converted to apparent ages using isotope-specific decay equations.

Underground production of ^36^Cl represents an additional uncertainty. Froehlich et al.^14^ estimated *in situ* production rates in the MRA of 4–6 × 10^–15^ and suggested that neglecting this contribution could increase apparent ^36^Cl ages by up to 30%. While non-negligible, this effect is modest relative to the magnitude of the diffusion-driven signal evident in both observations and simulations. This further supports the implicit consideration of underground production in the numerical model, as reflected in variability in initial ^36^Cl/Cl ratios, rather than being explicitly simulated.

For ^81^Kr, the extended model trend broadly envelopes the observed apparent ages but does not fully collapse their scatter. This residual variability likely reflects hydraulic heterogeneity, localized mixing, and the comparatively weaker influence of diffusion on noble gases. Overall, the results demonstrate that diffusion dominates ^36^Cl behavior at large distances, whereas ^81^Kr retains greater sensitivity to flow structure and residence time.

### Model Imitations

The modeling framework necessarily involves simplifications. Subsurface production of ^36^Cl and ^81^Kr was not explicitly simulated. Available estimates indicate that *in situ* production of ^81^Kr becomes significant only in uranium-rich lithologies,^32^ which are not representative of the investigated part of the MRA, though they may occur in the western part of the system, as indicated by the outlier in Figure 2. For ^36^Cl, previous studies have shown that *in situ* production is low with a secular equilibrium ^36^Cl/Cl ratio of 4–6 × 10^–15^ (Table 1).^14,151^ Uncertainties related to underground production were accounted for indirectly through sensitivity analyses of the initial ^36^Cl/Cl ratio rather than by explicit source-term simulation. Biogeochemical reactions affecting ^14^C, including carbonate dissolution–precipitation and isotopic exchange with dissolved inorganic carbon, were not included. This simplification is justified because ^14^C activities in the MRA are at or beyond the effective dating limit across most of the aquifer, and corrected ^14^C ages are not used for quantitative interpretation; omitting detailed carbon-system reactions therefore does not affect the conclusions based on longer lived tracers. Cross-formational flow and leakage through overlying formations, which have been invoked in conceptual models of the MRA,^12^ are not explicitly resolved. Finally, the confining units are treated as a single diffusive layer with uniform properties, although asymmetry between the upper and lower aquitards may influence tracer distribution.^59^ These simplifications do not alter the qualitative conclusions on the role of diffusion in controlling chlorine isotope systematics and the relative robustness of ^81^Kr, but they do contribute to quantitative uncertainty in inferred residence times.

### Implications for Applications of ^81^Kr and ^36^Cl to Date Old Groundwaters

The patterns observed in the MRA have broader implications for interpreting apparent ^81^Kr and ^36^Cl ages in other large fossil aquifer systems, such as the Continental Intercalaire in North Africa,^46^ the Nubian Sandstone Aquifer System,^16,47,153,154^ and the Great Artesian Basin in Australia.^17,45,145,155^

In many large fossil aquifer systems, ^36^Cl has been widely used to infer residence times on 10^5^–10^6^ year timescales. However, it has long been recognized that the interpretation of ^36^Cl data is particularly challenging when groundwater chloride concentrations vary, e.g., due to diffusive input of chloride from aquitards.^46^ In contrast, ^81^Kr has often been assumed to behave conservatively, including with respect to diffusive exchange.

Our results provide a quantitative assessment of this assumption in a system demonstrably affected by diffusion, as evidenced by the ^36^Cl systematics.

We find that the influence of diffusive exchange on ^81^Kr is minor relative to its impact on ^36^Cl, largely because krypton concentration gradients between the aquifer and surrounding formations are much smaller than those of chloride. For ^81^Kr, the activity ratio between the diffusion-free piston-flow scenario (pure radioactive decay) and the diffusion-corrected HGS model output is approximately 1.2, corresponding to only a 20% difference at the activity level (Figure 5a). When expressed in terms of apparent age, this translates into an overestimation of ^81^Kr age by a factor of ∼1.6 if diffusive exchange is neglected. In other words, ignoring diffusion would lead to apparent ^81^Kr ages that are up to 60% too old in the most downgradient part of the aquifer, whereas the effect is minor near recharge. This numerically derived age bias is consistent with the analytical steady-state solution of Sudicky and Frind (eq 13),^52^ which yields an identical age ratio of 1.6. A first-order estimate of this bias can also be obtained from the ratio of the total tracer-accessible pore volume to the mobile aquifer pore volume

5 $$R≅\frac{an_{\mathrm{aquitard}}+2Ln_{\mathrm{aquitard}}}{an_{\mathrm{aquifer}}}$$

where *a* is the aquifer thickness (90 m), *n* denotes porosity of the aquifer and aquitards (10 and 7%, respectively), and *L* is the diffusion length scale (eq 6).

6 $$L≅\sqrt{\frac{D_{\mathrm{eff}}}{\lambda }}$$

Taken together, these results demonstrate that ^81^Kr provides a more robust primary chronometer for very old groundwater, whereas ^36^Cl and stable chlorine isotopes are better suited as tracers of solute sources and transport processes.^15,16,35,45,47,84^ In systems bounded by thick aquitards or evaporite-rich confining units, our findings therefore support the joint application of ^81^Kr, ^36^Cl/Cl, and δ^37^Cl, combined with explicit advection–diffusion modeling, to distinguish true groundwater residence times from artifacts introduced by diffusive solute exchange.

Although groundwater “age” does not represent a single physical time since recharge, mean residence times and age distributions are essential diagnostics of aquifers across arid and semi-arid regions worldwide. They constrain recharge rates, flow velocities, and the balance between renewable inflow and long-term storage. In regional fossil aquifers such as the MRA, residence times on the order of 10^5^–10^6^ years indicate that most extracted water derives from finite storage rather than modern recharge, implying recovery times that far exceed management horizons. Age information also helps to identify zones where younger recharge or cross-formational inflow contributes to pumped water, which is critical for assessing vulnerability, water-quality evolution, and the long-term sustainability of groundwater use. By distinguishing between true residence time and apparent ages biased by transport processes, the integrated tracer–modeling approach developed here provides a sound basis for interpreting groundwater age and recharge histories in other major fossil groundwater reservoirs that underpin water supply across arid and semi-arid areas.

## Conclusion

This study combines new tracer measurements with numerical flow-and-transport simulations to reassess groundwater residence times and solute transport processes in the Milk River Aquifer. The results provide clear evidence that diffusive exchange between the aquifer and adjacent aquitards is a major control on the distribution of chloride, ^36^Cl/Cl ratios, and δ^37^Cl values along the regional flow path. The model reproduces the observed decline in ^36^Cl/Cl ratios and the systematic increase in δ^37^Cl values using plausible ranges of transport and boundary-condition parameters, indicating that the diffusive influx of chloride from Cl-rich, ^36^Cl-depleted aquitard water dominates the tracer evolution. The formation of a “halo” with elevated δ^37^Cl values at aquifer margins is consistent with diffusion-driven isotopic fractionation documented in laboratory and natural systems, reinforcing the value of δ^37^Cl as an indicator of long-term solute exchange across low-permeability boundaries.

The same model, adjusted with transport parameters appropriate for Kr, also agrees qualitatively with the new ^81^Kr observations and indicates a limited sensitivity to diffusive effects. Finally, the close agreement between the explicit numerical simulations presented here and the analytical solutions of Sudicky and Frind^52^ demonstrates that these simplified formulations provide a reliable, and now empirically supported, means of estimating the effective retardation of ^81^Kr in double-porosity systems such as the MRA.

The agreement between simulated and observed trends across multiple tracers supports existing conceptualizations of the MRA as a low-recharge, long-residence-time groundwater system. At the same time, the results highlight the need for careful interpretation of ^36^Cl/Cl in diffusion-dominated settings, where apparent ages reflect both decay and the addition of external chloride sources. Further refinements, such as explicit simulation of *in situ* radionuclide production, biogeochemical reactions affecting ^14^C and δ^13^C, and cross-formational leakage, would help reduce remaining uncertainties and better constrain tracer systematics.

Overall, this study provides a quantitative basis for evaluating the sensitivity of ^81^Kr/Kr and ^36^Cl/Cl ratios to diffusive processes in regional aquifers. The findings underscore the advantages of a multitracer approach and provide a framework to support future investigations of other large fossil groundwater systems. Integrating these chronometers with paleo-hydrological reconstructions represents a promising direction for future work, particularly for clarifying how Late Pleistocene climate variability influenced recharge and groundwater storage in continental-scale aquifers.

## Supplementary Material



## Notes

Figure illustrating the HGS model domain, hydraulic head boundary conditions, and resulting flow velocities (PDF)

## ○

Reika Yokochi sadly passed away in February 2024.
