# Supplementary material for: Diffusion-Controlled Solute and Isotope Transport in the Milk River Aquifer System, Alberta, Canada: Implications for Dating Old Groundwater
Source: ACS Earth Space Chem. 2026 Apr 9;10(5):1291–309. doi: 10.1021/acsearthspacechem.5c00397 (PMC13200262; doi:10.1021/acsearthspacechem.5c00397)
Supplement: Supplementary file 1 [file sp5c00397_si_001.pdf]

Diffusion-controlled Solute and Isotope Transport in the Milk River Aquifer System, Alberta, Canada: Implications for dating old groundwater

Stephanie L. Musy[1,2], Roland Purtschert[2], Neil C. Sturchio[3], Linnea J. Heraty[3], Peter Mueller[4], Jeremy Lantis[4], Michael N. Bishof[4], Christof Vockenhuber[5], Avadhoot Date[6], Bernhard Mayer[6], Reika Yokochi[7†]*

[1]Hydrogeology, Environmental Sciences, University of Basel, 4056 Basel, Switzerland

[2]Climate and Environmental Physics, University of Bern, 3012Bern, Switzerland

[3]Earth Sciences, University of Delaware, Newark, Delaware, USA

[4]Argonne National Laboratory, Lemont, Illinois, USA

[5]Laboratory of Ion Beam Physics, ETH Zurich, 8092 Zurich, Switzerland

[6]Department of Earth, Energy, and Environment, University of Calgary, Calgary, Alberta, T2N 1N4, Canada

[7]Geophysical Sciences, University of Chicago, Chicago, Illinois, USA

HydroGeoSphere Model

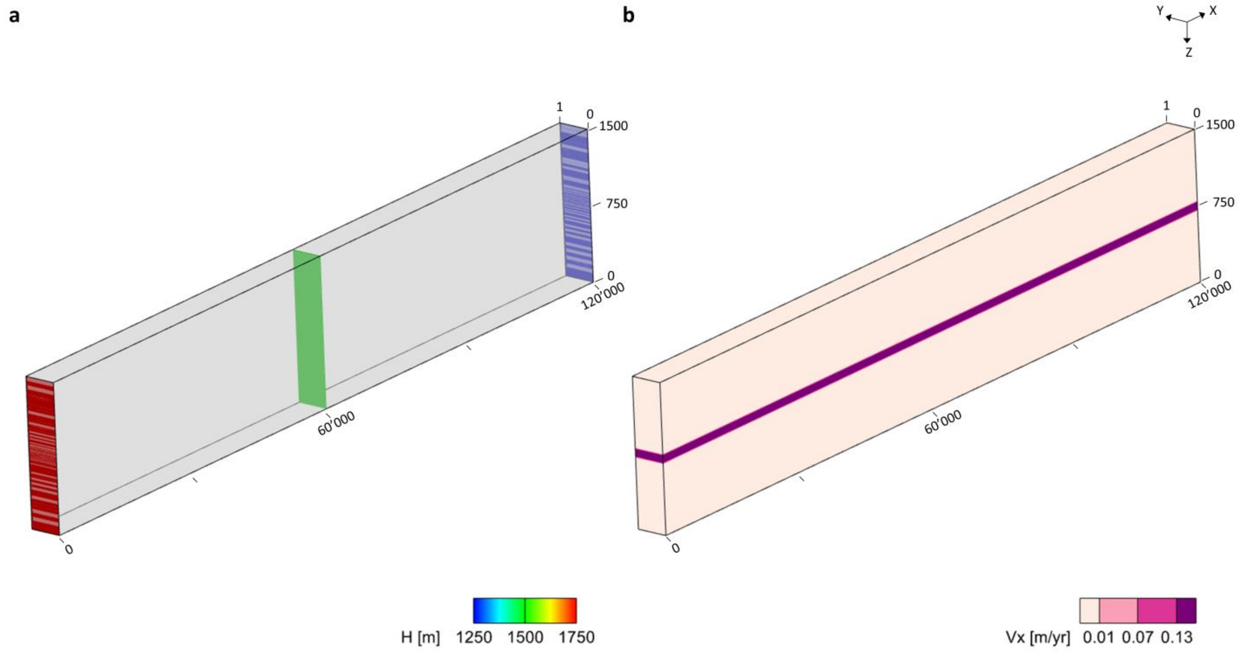

Figure S1: (a) Schematic representation of the 2D model domain, illustrating an idealized vertical cross-section of the Milk River Aquifer (MRA) and the overlying and underlying aquitard formations. Colored surfaces indicate the imposed hydraulic head boundary conditions; (b) Simulated distribution of horizontal groundwater velocity (Vx), showing negligible flow in the aquitards and a peak velocity of approximately 13 cm yr^{-1} within the aquifer. Distances are in meters.
